# Editorial: Hybrid (combined endovascular and microsurgical) treatments for cerebrovascular diseases

**DOI:** 10.3389/fneur.2024.1378269

**Published:** 2024-03-11

**Authors:** Basil E. Grüter, Davide Croci, Kunal Vakharia, Philipp Gruber, Philipp Taussky

**Affiliations:** ^1^Institute of Neuroradiology, Aarau Cantonal Hospital, Aarau, Switzerland; ^2^Service de Neuroradiologie, Hôpital de la Timone, Marseille, France; ^3^Department of Neurosurgery and Brain Repair, University of South Florida, Morsani College of Medicine, Tampa, FL, United States; ^4^Beth Israel Deaconess Medical Center, Harvard Medical School, Boston, MA, United States

**Keywords:** hybrid operation room, endovascular treatment, microsurgical treatment, cerebrovascular disease, intracranial aneurysm, dural fistula, arterio-venous malformation (AVM)

Most cerebrovascular diseases can effectively be treated with microsurgical operations, endovascular procedures or radiation therapy. In some cases, a combination of these techniques may be necessary, either in a staged workflow or simultaneously in a hybrid operation. Technical progress during the last several decades has led to the construction of hybrid operation rooms – combining microsurgical and endovascular facilities in many neurovascular units all over the globe. A classic neurovascular hybrid operation is the surgical exposure of an otherwise inaccessible blood vessel for direct cannulation and endovascular treatment (1, 2). A different form of a hybrid operation is a direct intraoperative control-angiography after microsurgical clipping, which aids in detecting remnants (3) or periprocedural vasospasm (4). However, in such a hybrid approach, the purpose of the endovascular part remains purely diagnostic.

In select cases, a multimodality approach may be needed utilizing both endovascular and microsurgical techniques. For instance, an endovascular aneurysm treatment combined with minimally invasive endoscopic hematoma evacuation (5) or combined clipping/coiling procedures can be used to streamline treatment of a ruptured aneurysm with significant mass effect from the hematoma (6, 7). In addition, microsurgical creation of an extra-intracranial bypass in combination with endovascular aneurysm obliteration may be an alternative for more complex morphologies (8–11). In line with these studies Rennert et al. reports his series of 10 patients with complex, mostly ruptured aneurysms that underwent combined open revascularization (intra-intra or extra-intracranial bypass) and endovascular aneurysm embolization. These cases were not amenable to stand-alone open or endovascular procedures. The combined hybrid approach allowed the authors to recognize and preserve perforator arteries. Many of these modalities focus on unique aspects of either endovascular or microsurgical techniques. There are some hybrid options that utilize some of the endovascular armamentarium in unique ways. A different from of a true hybrid approach for treatment of aneurysms is to achieve proximal control by means of an endovascular ballon occlusion and treat the aneurysm with microsurgical clipping. This has also been highlighted by the Dallas technique in which balloon occlusion proximally can allow for modulation of aneurysmal dome to allow for easier microsurgical access (12). Similar techniques have also previously been described for some posterior circulation aneurysms (13). The work of Zhang et al. focused on giant paraclinoid aneurysms. They refined the technique and suggested to inject methylene blue into the balloon, in order to improve its visibility, optimize its positioning, and avoid excess radiation exposure.

Although complex aneurysm management is one of the key uses of hybrid cerebrovascular surgery, there are also potential benefits when treating other pathologies including steno-occlusive disease and vascular malformations. The feasibility and potential beneficial effects of presurgical embolization of high-grade arterio-venous malformations (AVMs) in a hybrid operation has been shown several times (14–16). Highly select complex dural fistulas may be treated with a true hybrid approach, if a single modal therapy failed or does not seem to be sufficiently promising (17). Xiao et al. present their series foramen magnum fistulas, underscoring that even in this pathology, there are a small subset that require a hybrid approach.

The optimal treatment strategy for carotid artery stenosis remains controversial (18, 19). Cai et al. share their experience with a hybrid approach (carotid endarterectomy combined with carotid stenting) for complex chronic internal carotid artery occlusion. In their experience, they found revascularization of occluded carotid arteries was safe and feasible and correlated with whether the occluded segment extended past the ophthalmic artery. Similar work in steno-occlusive disease done by Kuo et al. demonstrate a compelling case of a life-threatening innominate artery dissection treated with simultaneous placement of two kissing stents. While one stent was delivered via a percutaneous retrograde endovascular route through the right brachial artery, the second one required a combined hybrid approach, involving open surgical distal clamping of the common carotid artery, and a retrograde endovascular transcarotid approach.

Further articles in this Research Topic cover very rare, interesting cerebrovascular pathological conditions but are not directly related to a therapeutic hybrid approach. As such, Zeng et al. report a *de-novo* formation of an extracranial aneurysm after endovascular embolization of a vertebral epidural arterio-venous fistula in a patient with underlying neurofibromatosis I. The case report of Xu and Li is on the formation of a pseudoaneurysm of the middle meningeal artery after craniotomy with consecutive successful endovascular treatment. Yu et al. reports a study on patients with hypertensive brainstem hemorrhage and found that hematoma volume correlated with the 30-days mortality in these patients. Lastly, with the aim of an optimal patient's monitoring during the operation, Tang et al. present their experience with intraoperative neurophysiological monitoring (MEP/SSEP) during microsurgical clipping of posterior communicating aneurysms, particularly for monitoring the patency and vasospasm of the adjacent anterior choroidal artery.

In summary, the combination of endovascular and microsurgical strategies in a single hybrid operation is both feasible and safe. This approach can address various cerebrovascular disease and its necessity arises in selective complex cases, demanding a sophisticated infrastructure and a high level of specialization and expertise for which we have to train the next generation.

## Author contributions

BG: Conceptualization, Project administration, Validation, Writing – original draft, Writing – review & editing. DC: Project administration, Writing – review & editing. KV: Project administration, Writing – review & editing. PG: Project administration, Writing – review & editing. PT: Project administration, Supervision, Writing – review & editing.
